# Transient Receptor Potential Melastatin 2 Negatively Regulates LPS-ATP-Induced Caspase-1-Dependent Pyroptosis of Bone Marrow-Derived Macrophage by Modulating ROS Production

**DOI:** 10.1155/2017/2975648

**Published:** 2017-11-08

**Authors:** Haihong Wang, Xinyi Zhou, Hui Li, Xiaowei Qian, Yan Wang, Liang Ma

**Affiliations:** ^1^Department of Anesthesiology, Sir Run Run Shaw Hospital, School of Medicine, Zhejiang University, Hangzhou 310016, China; ^2^Department of Anesthesiology, The Third Affiliated Hospital, Anhui Medical University, Hefei 230061, China; ^3^Department of Anesthesiology, The First Affiliated Hospital, School of Medicine, Zhejiang University, Hangzhou 310003, China; ^4^Department of Anesthesiology, Women's Hospital, School of Medicine, Zhejiang University, Hangzhou 310006, China; ^5^Department of Urology, Sir Run Run Shaw Hospital and the Institute of Minimally Invasive Surgery, School of Medicine, Zhejiang University, Hangzhou 310016, China

## Abstract

**Background:**

Pyroptosis, a new form of cell death, which has special morphological characteristics, depends on caspase-1 activation and occupies an important role in inflammatory immune diseases and ischemia-reperfusion injury. ROS is a common activator of NLR/caspase-1. Transient receptor potential melastatin 2 (TRPM2), a selective cation channel, is involved in inflammatory regulation. This study was designed to explore the role of TRPM2 in activating caspase-1 and caspase-1-dependent pyroptosis of mouse BMDMs.

**Methods:**

BMDMs isolated from WT and TRPM2−/− mice were treated with LPS and ATP, along with ROS inhibitor (NAC and DPI), caspase-1 inhibitor (Z-YVAD), or not. The activation of caspase-1 was measured by western blot. EtBr and EthD-2 staining were used to assess the incidence of pyroptosis.

**Results:**

Compared with WT, the activated caspase-1-P10 was higher and the percentage of EtBr positive cells was also increased in TRPM2−/− group, which were both inhibited by Z-YVAD, NAC, or DPI. ASC oligomerization was increased in TRPM2−/− group.

**Conclusion:**

Deletion of TRPM2 can enhance the activation of caspase-1 and pyroptosis, which may be via modulating ROS production, suggesting that TRPM2 plays a critical role in immune adjustment.

## 1. Introduction

Sepsis is regarded as the most common cause of death in intensive care unit, which is typically characterized by initial cytokine storm and a subsequently significant immunosuppression [1, 2]. The proinflammatory cytokines including IL-1*β*, IL-6, IL-18, and TNF-*α* have been reported to contribute to initial fatal systemic inflammatory response syndrome in the early stage of sepsis [1, 3, 4]. Previous studies demonstrate that immune paralysis of sepsis was correlated with activation of suppressive receptors, induction of inhibitory ligands on immune cells, and expansion of suppressive cell types [5–7]. Recently, studies have shown that cell death-induced depletion of immune cells in sepsis plays a vital role in immunosuppression [8–10].

Pyroptosis, a supramolecular assembly of ASC dimers, is a recently identified kind of cell death and dependent on caspase-1 [10–12]. During the process of pyroptosis, the membrane pores between 1.1 and 2.4 nm in diameter are first generated, followed by water uptake and cell swollen by dissipated ionic gradients, and cells ultimately underwent osmotic lysis with release of intracellular contents [13]. It has been demonstrated that pyroptosis has an important role in inflammatory immune diseases, and reactive oxygen species (ROS), K^+^ efflux, and phagosomal destabilization are involved in the mechanism of caspase-1-dependent pyroptosis [14, 15].

Transient receptor potential melastatin 2 (TRPM2), an oxidant-sensitive and Ca^2+^ permeable nonselective cation channel, is highly expressed in immunocytes including monocytes, macrophages, and lymphocytes [16]. TRPM2 can be opened through directly binding with intracellular adenosine diphosphate ribose (ADPR) and also be indirectly activated under conditions of oxidative stress, acidification, and elevated intracellular Ca^2+^ [17–19]. Meanwhile, the activation of TRPM2 can reduce NADPH oxidase-activated ROS production [20]. Studies have been performed on the effect of TRPM2 in the pathogenic processes of inflammation, ischemia-reperfusion injury, diabetes, and neurodegenerative disorders [16]. However, no study has been done to evaluate the relationship between TRPM2 and caspase-1-dependent pyroptosis. Thus, in this study, we aim to investigate the role of TRPM2 in regulating caspase-1-dependent pyroptosis of bone marrow-derived macrophage in vitro and its underlying mechanism.

## 2. Materials and Methods

### 2.1. Animal Preparation

Male C57BL/6 mice (6–8 weeks) were purchased from Laboratory Animal Center (Chinese Academy of Sciences, Shanghai). TRPM2-KO mice were provided by Professor Y. mori (Graduate School of Engineering, Kyoto University, Kyoto, Japan). The mice were maintained in a specific pathogen-free facility with ad libitum access to food and water. Animal experiments were approved by the Committee on the Use of Live Animals in Teaching and Research from ZJU.

### 2.2. Cell Isolation and Culture

After mice were sacrificed by cervical dislocation, bone marrow-derived macrophage (BMDM) was flushed from mouse femurs and tibias with sterile RPMI 1640 medium (Invitrogen) and subsequently depleted of red blood cells using ammonium chloride. Then, the cells were cultured in RPMI 1640 supplemented with 10% FBS, 0.1 mM nonessential amino acids, 2 mM L-glutamine, 1 mM sodium pyruvate, 100 units/ml penicillin, 100 *μ*g/ml streptomycin, and 20 ng/ml mouse M-CSF (PeproTech) for 5–7 days at 37°C under 5% CO_2_. Nonadherent cells were carefully removed, and fresh medium was replaced every 3 days. Adherent cells > 98% were judged as available macrophages by morphology. Macrophages were replated onto 6-well plates at 2 × 10^6^ cells per well or seeded in 24-well plates at 5 × 10^5^ cells per well and used for experiments within 24 to 48 h. Unless otherwise indicated, macrophages were primed with 1 ug/ml LPS from* Escherichia coli* 0111:B4 (Sigma) for 4 h followed by stimulation with 5 mM ATP for 30 min. For pharmacological assessments, some samples were subsequently treated with 10 mM NAC (Sigma), 12.5 uM DPI (Sigma), 5 uM Z-YVAD (ENZO), or 130 mM KCl, respectively, to the cell culture for 30 min before stimulation with ATP.

### 2.3. Western Blotting Analysis

Cells were lysed in Radio Immunoprecipitation Assay (RIPA) lysis buffer containing protease inhibitors phenylmethylsulfonyl fluoride (PMSF), followed by incubation on ice for 40 min with vigorously shaking every 10 minutes. After centrifugation at 12,000 rpm for 10 min, each clarified lysates were subjected to 12% SDS-PAGE gels and transferred onto nitrocellulose membranes (Bio-Rad). Membranes were blocked in TBS-T containing 5% bull serum albumin (BSA, Gibco) for 1 hour. The total amount of caspase-1 p-10 was detected by rabbit polyclonal antibody against mouse caspase-1 p-10 antibody (sc-514, Santa Cruz). Anti-*β*-actin antibody was diluted to 1 : 2000 (Sigma). After incubating overnight, membranes were washed three times in clear TBS-T for 10 min and incubated by anti-rabbit horseradish peroxidase-conjugated secondary antibody (Sigma) for 1 hour. Membranes were rinsed three times in TBS-T for 10 min, then blots were visualized by EZ-ECL (Biological Industries) and imaged by X-ray film.

### 2.4. ASC Pyroptosome Detection

Macrophages were seeded in 6-well plates (2 × 10^6^ cells per well) and treated with different stimuli. The culture supernatants were precipitated and analyzed by immunoblotting of caspase-1 p20 using polyclonal rabbit anti-human ASC (SC-22514-R, Santa Cruz) as described above. The cells pellets were harvested and then lysed in buffer A (20 mM Hepes-KOH, pH 7.5, 10 mM KCl, 1.5 mM MgCl_2_, 1 mM EDTA, and 1 mM EGTA) containing 0.1 mM PMSF and protease inhibitor mixture. The solution was diluted with one volume CHAPS buffer (20 mM Hepes-KOH, pH 7.5, 5 mM MgCl_2_, 0.5 mM EGTA, 0.1 mM PMSF, and 0.1% CHAPS) and then centrifuged at 6000 rpm in 1.5 ml Eppendorf tubes to pellet ASC pyroptosomes. After discarding the supernatants, the resultant pellets were resuspended in 500 ul CHAPS buffer per tube. The resuspended pellets were crosslinked with 4 mM fresh disuccinimidyl suberate (DSS) cross-linker (Sigma) for 30 minutes at 37°C with flipping the tubes every 15 min and then pelleted by centrifugation at 6000 rpm for 10 min. The crosslinked pellets were resuspended in 30 *μ*l of SDS sample buffer, fractionated on 12% SDS-PAGE, and analyzed by western blotting using anti-mouse ASC antibodies mentioned above.

### 2.5. Ethidium Bromide (EtBr) and Ethidium Homodimer-2 (EthD-2) Staining

Macrophages were seeded in 24-well plates (5 × 10^5^ cells per well) and treated with different stimuli. The cells were gently washed twice with PBS and fixed by 4% paraformaldehyde for 10 min. After washing the cells once again and adding 200 ul PBS per well, cells were stained with 4,6-diamidino-2-phenylindole (DAPI, 0.1 ug/ml) (Vector Laboratories) for 15 min. Instantly after adding red membrane impermeant dyes, either EtBr (25 *μ*g/ml, Sigma) or EthD2 (25 *μ*g/ml, Life Technologies) was added at 0.5 ul per well. The cells were observed immediately after adding EtBr or EthD2 and analyzed using confocal microscope (Olympus). The percentage of positive cells was counted and calculated at an amount of at least 300 cells per group for each experimental condition.

### 2.6. Statistical Analysis

The data were reported as mean ± SD from three independent experiments using SPSS16.0 for Windows (SPSS, Inc., Chicago, IL) unless otherwise noted. Student's *t*-test was used for statistical analyses between two groups and multiple different comparisons were performed by using one-way ANOVA test. *P* value less than 0.05 was considered statistically significant, and *P* < 0.001 was highly significant.

## 3. Results

### 3.1. The Activation of Caspase-1 Was More Prominent in BMDM of TRPM2−/− Group

**Figure 1:**
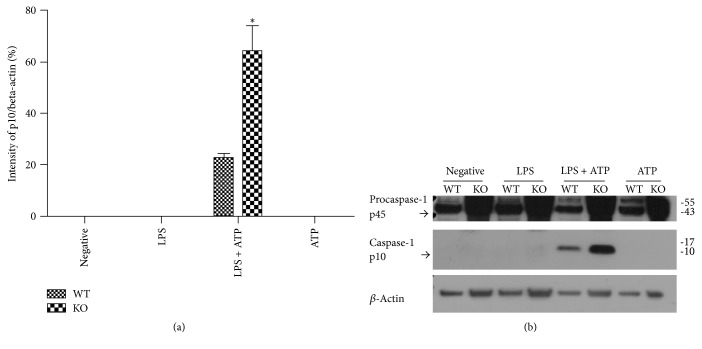
The activated caspase-1-P10 level in BMDM of TRPM2 KO mice and WT mice. *∗* means that TRPM2−/− group compared to WT group. The activated caspase-1-P10 level was significantly higher in TRPM2−/− group compared with WT group.

To explore the activation degree of caspase-1 in TRPM2−/− mice, BMDM was treated with 1 *μ*g/mL LPS for 4 h followed by 5 mM ATP for 30 min to induce caspase-1 activation, which was tested by western blotting. Only 1 *μ*g/mL LPS plus 5 mM ATP treatment activated caspase-1 in BMDM of WT and TRPM2−/− mice, and the activated caspase-1-P10 level was significantly higher in TRPM2−/− group compared with WT group (Figure 1).

### 3.2. Caspase-1-Dependent Pyroptosis Was Increased in TRPM2−/− Group Compared to WT Group

**Figure 2:**
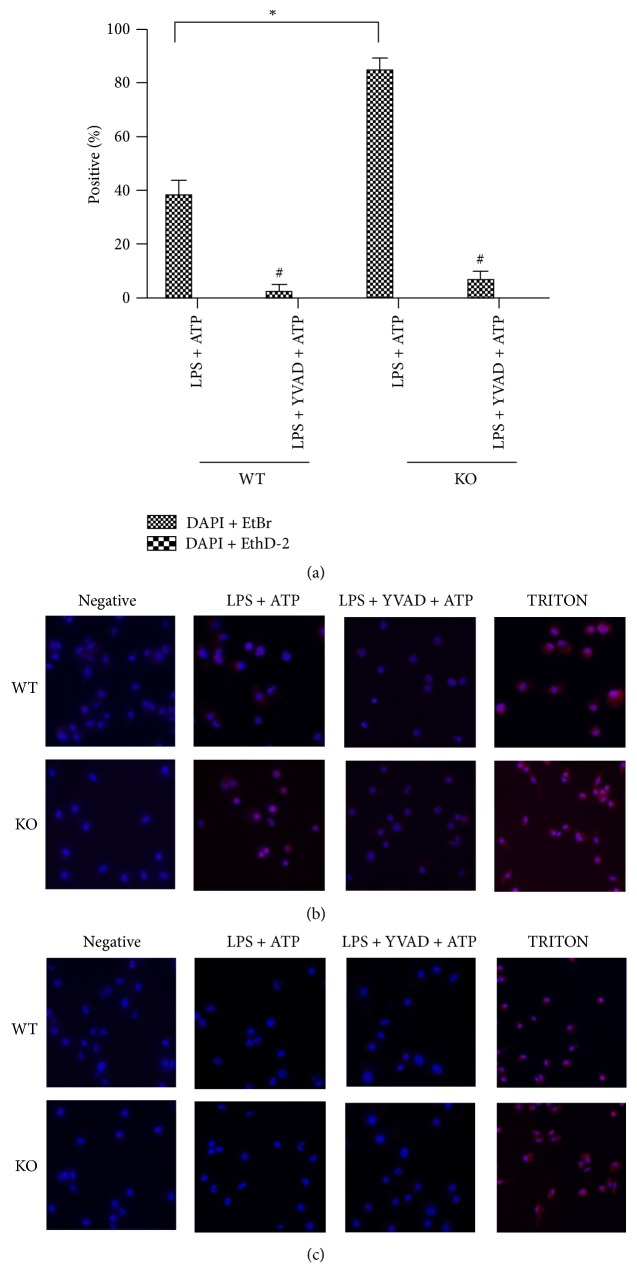
Caspase-1-dependent pyroptosis was increased in TRPM2−/− group compared to WT group. *∗* means that TRPM2−/− group compared to WT group; # means that LPS + YVAD + ATP compared to LPS + ATP in the same type mice. The percentage of EtBr positive cells was significantly increased in the TRPM2−/− group compared to WT group after LPS plus ATP treatment, but the percentage of EthD-2 positive cells was similar between two groups.

The formation of pore with diameter of 1.1–2.4 nm occurs in cell membrane during the process of pyroptosis. EtBr with low molecular weight (MW 394 Da) can go through such pore but EthD-2 with high molecular weight (MW 1293 Da) cannot. Therefore, EtBr and EthD-2 staining positive cells can reflect the occurrence of pyroptosis. We used small membrane impermeant dye EtBr, larger EthD-2, and the membrane permeable dye DAPI to examine the formation of pore with diameter of 1.1–2.4 nm in cell membrane during pyroptosis. Triton is a liposoluble active agent that can dissolve the lipid of cell membrane and nuclear membrane, thus forming a larger hole to make all dye enter. So triton can be regarded as a positive control. We found that the percentage of EtBr positive cells was significantly increased in the TRPM2−/− group compared to WT group after LPS plus ATP treatment, but there were almost no EthD-2 positive cells in both groups. In addition, the percentage of EtBr positive cells in WT and TRPM2−/− group was reduced by caspase-1 inhibitor (Z-YVAD) (Figure 2).

### 3.3. In TRPM2−/− Mice, the Activation of Caspase-1 and Caspase-1-Dependent Pyroptosis Was Inhibited by NAC Treatment or DPI Treatment

**Figure 3:**
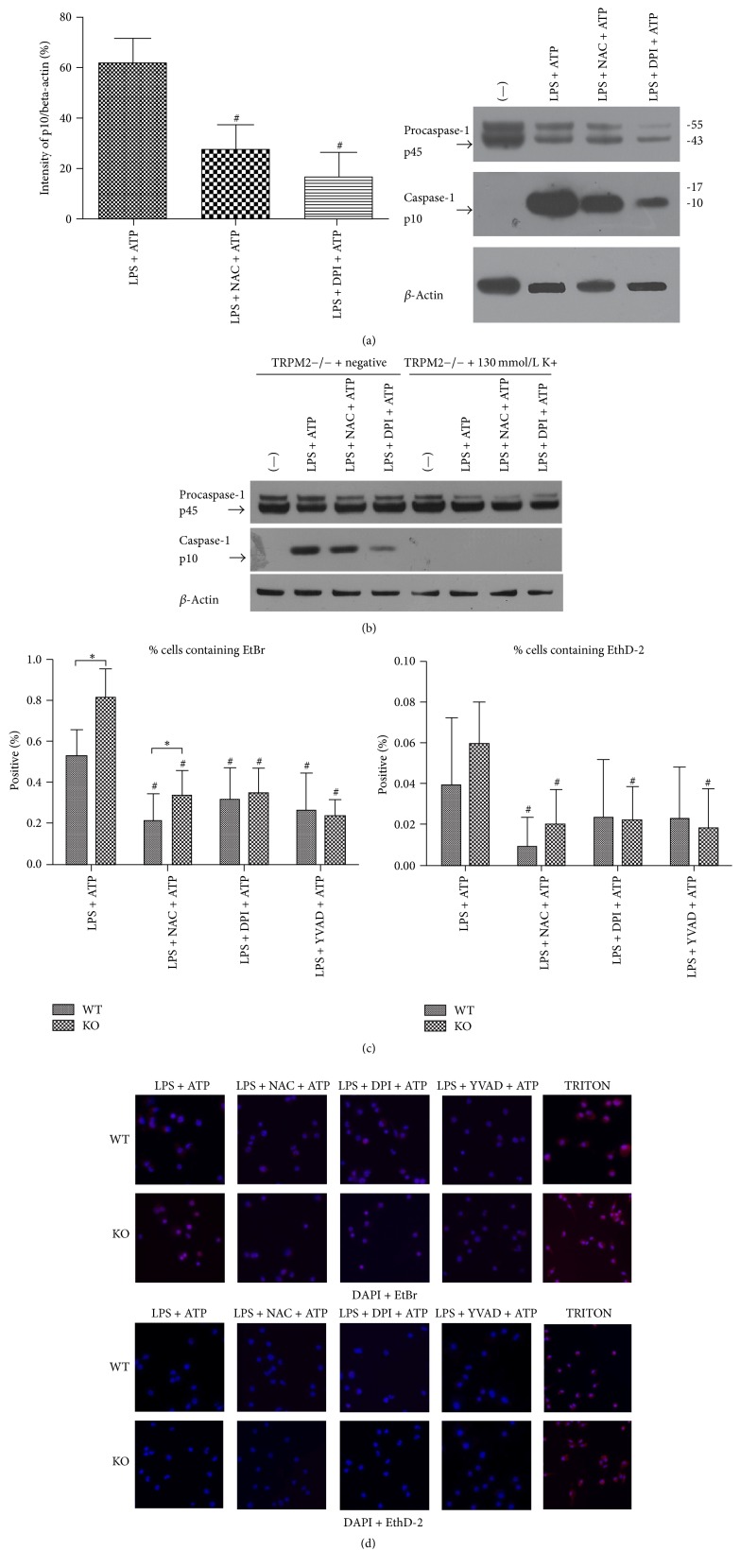
In TRPM2−/− mice, the activation of caspase-1 and caspase-1-dependent pyroptosis was inhibited by NAC treatment or DPI treatment. # means that this group compared to LPS + ATP group in the same type mice. *∗* means that TRPM2−/− group compared to WT group. In TRPM2−/− mice, the activation of caspase-1 was reduced in LPS + NAC + ATP group and LPS + DPI + ATP group compared to LPS + ATP group.

Both caspase-1 and caspase-1-dependent pyroptosis were more prominent in TRPM2−/− mice. Since ROS production and K+ efflux are common steps of NLR/caspase-1 complex activation, we hypothesized that TRPM2 negatively regulates LPS-ATP-induced caspase-1 and caspase-1-dependent pyroptosis through modulating ROS production and K+ efflux. So we treated BMDM of TRPM2−/− mice with ROS inhibitor DPI or NAC (ROS scavenger) or DPI (NADPH oxidase inhibitor) before ATP stimulation and added 130 mmol/L K^+^ into medium before LPS and ATP stimulation. As predicted, in TRPM2−/− mice, the activation of caspase-1 was reduced in LPS + NAC + ATP group and LPS + DPI + ATP group compared to LPS + ATP group (Figure 3(a)).

Previous study has found that high extracellular potassium concentration (130 mM) can block the activation of NALP3 inflammasome and inhibit the activation of caspase-1. In our study, in the condition of 130 mmol/L extracellular K^+^, caspase-1 activation was disappeared, but procaspase-1 was not affected (Figure 3(b)).

The percentage of EtBr positive cells was significantly reduced by DPI or NAC treatment but the percentage of EthD-2 positive showed no significant difference (Figures 3(c)-3(d)).

### 3.4. ASC Oligomerization Was Largely Increased in TRPM2−/− Group Compared to WT Group

**Figure 4:**
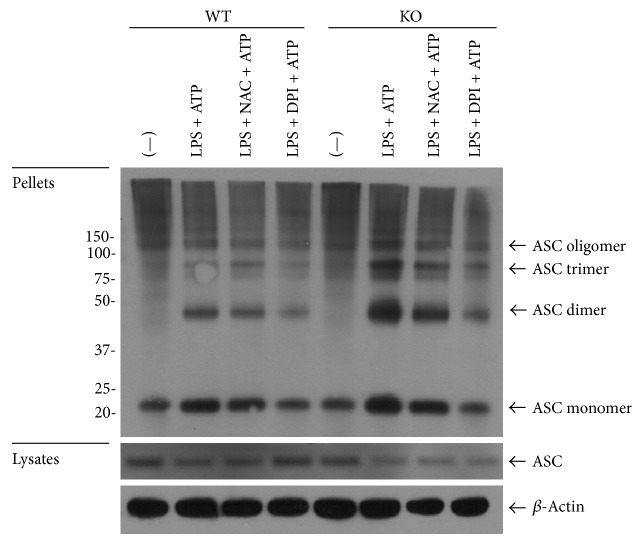
ASC oligomerization was largely increased in TRPM2−/− group compared to WT group. ASC oligomerization was significantly higher in TRPM2−/− group compared to WT group. And in both TRPM2−/− group and WT group, ASC oligomerization was inhibited by NAC or DPI treatment.

ASC oligomerization is an objective index of pyroptosis. DSS can make chemical crosslinking between NLRP3 mediated ASC dimer, trimer, and oligomer that can be separated and by 5000 ×g low-speed centrifugation. We used western blotting to detect the degree of ASC oligomerization. ASC oligomerization was significantly higher in TRPM2−/− group compared to WT group. And in both TRPM2−/− group and WT group, ASC oligomerization was inhibited by NAC or DPI treatment (Figure 4).

## 4. Discussion

This study demonstrated that TRPM2 can negatively regulate the LPS-ATP-induced caspase-1-dependent pyroptosis in bone marrow-derived macrophage and the possible mechanism might be related to the modulation of ROS production.

As we know, TRPM2 has a protective role in the regulation of inflammatory disorders/immune disease. Interleukin 6 (IL-6), macrophage inflammatory protein 2 (MIP-2), and tumor necrosis factor (TNF) were increased in the lungs of TRPM2 KO mice, which suggested the protective role of TRPM2 in lung inflammation [20]. In addition, TRPM2 channels preserved mitochondrial biological function and further protected the hearts which suffered from ischemia-reperfusion injury [21]. However, the exact mechanism of TRPM2 in immune regulation is still elusive. Our study provides potential evidence that TRPM2 may modulate caspase-1-dependent pyroptosis in inflammatory immune diseases.

To the best of our knowledge, pyroptosis is a double-edged sword in the progress of sepsis. On one hand, pyroptosis is advantageous for host defense against intracellular pathogens by eliminating replicative niche, releasing the intracellular pathogens into the extracellular space, which is convenient for neighboring phagocytes to uptake and kill them as a result. On the other hand, pyroptosis is important in the inflammatory process since it can make activated macrophages rapidly release large amounts of cytokines into the extracellular space, and pyroptosis-induced depletion of immune cells is associated with cytopenia and immune suppression [22–24]. Therefore, we speculate depletion of immune cells and inflammation promotion have the dominant role in LPS-ATP-induced pyroptosis of bone marrow-derived macrophage.

The question of how TRPM2 modulates LPS-ATP-induced pyroptosis of bone marrow-derived macrophage arises. By comparing the severity of mitochondrial dysfunction between WT group and TRPM2 KO group, Miller et al. postulate the protection of TRPM2 against hearts ischemia-reperfusion injury is due to ameliorated mitochondrial dysfunction and reduced ROS production [21]. However, ROS can be produced by several sources, including NADPH oxidase, mitochondria, and 5-lipoxygenase [25]. ROS is believed to be a common NLR/caspase-1 complex activator, and activated caspase-1 can mediate a large structure assembled by ASC, which is termed as pyroptosis [26]. The results in our study confirmed the involvement of ROS in the process of pyroptosis. Our result is similar with that from Di et al. who confirmed the mechanism of TRPM2 in downregulating ROS production, which is that TRPM2 increases membrane depolarization and thereby inhibits electrogenic activity of NADPH oxidase and blocks ROS production as a result [20].

It has been reported K+ efflux is necessary for caspase-1 activation induced by bacterial toxins and particulate matter [22]. So we treated LPS-ATP-stimulated BMDM of TRPM2−/− with 130 mmol/L K+ and found that there was no caspase-1 activation and pyroptosis happened. Therefore, we confirmed that K+ efflux acts on the upstream of caspase-1 and pyroptosis, and it is the requirement that TRPM2 regulates LPS-ATP-induced pyroptosis of bone marrow-derived macrophage.

## 5. Conclusions

Our study revealed that TRPM2 negatively regulates LPS-ATP-induced caspase-1-dependent pyroptosis of bone marrow-derived macrophage by modulating ROS production.
